# Lupus clinical trials and the promise of future therapies

**DOI:** 10.2478/rir-2023-0018

**Published:** 2023-09-27

**Authors:** Leila Khalili, Wei Tang, Anca D. Askanase

**Affiliations:** Columbia University Irving Medical Center, New York , NY, USA

Several successful phase 3 trials and the approval of these new therapies in systemic lupus erythematosus (SLE), have resulted in increased interest in drug development in lupus.^[1, 2, 3]^ Despite the recent approval of new therapies for SLE and lupus nephritis (LN) there is still an urgent need for advanced therapies as response rates are still in the 50%–60% range.^[4, 5, 6]^

Following the failure of two recently published phase 3 trials that failed to meet their endpoints, emphasis was refocused on data quality in lupus trials. ^[7, 8, 9]^ It followed that the efforts to adjudicate patients at entry in trials, adjudicate endpoints, and careful monitoring of disease activity instruments throughout the trial become the norm. While these changes are likely to provide better data in the upcoming trials, lupus investigators, are following with trepidation these large phase 3 development programs. The landscape of clinical trials in lupus is evolving. The current article will discuss the more recent failed phase 3 trials, several innovative phase 2 studies, and the new and exciting cell therapy data. Both phase 3 studies reviewed in this article were preceded by successful Phase 2 trials.

As it has become an alarming trend, two recent phase 3 clinical trials, that were preceded by successful phase 2 studies, failed to meet their primary endpoints. SLE clinical trials face multifaceted challenges at the patient, investigator, trial, study site, sponsor, and regulatory levels.

## Patient Level Challenges

SLE is a disease well known for heterogeneity in clinical presentation and pathophysiology. SLE manifestations vary and include a combination of constitutional, mucocutaneous, neuropsychiatric, musculoskeletal, cardiopulmonary, gastrointestinal, ophthalmologic, hematologic, and renal symptoms.^[10]^ A recent conceptual model proposed two groups of SLE symptoms; Type 1 inflammatory/immune mediated manifestations (arthritis, nephritis, rashes) and Type 2 symptoms like fatigue, myalgia, mood disturbance, cognitive dysfunction that are less likely to be autoimmune in nature.^[11]^ Furthermore, transcriptomic profiling revealed immunologic heterogeneity that can be categorized in four subgroups based on the main drivers the pathogenic process, type I interferon, neutrophil/myeloid cells, cytokine, and lymphocyte.^[10]^ Despite repeated confirmation of these transcriptomic data, biomarkers have not yet been used as inclusion criteria for SLE clinical trials. ^[12, 13, 14]^ Patients’ race and ethnicity add another layer of heterogeneity in lupus; SLE affects diverse population of patients; Black and Hispanic females have the highest incidence, prevalence and disease severity.^[15]^ In addition to heterogeneity, patient level challenges include the small number of eligible patients because of many competing studies and an increase in reluctance to participate in clinical trials.^[16,17]^

## Trial Design Challenges

Increasingly complex inclusion/exclusion criteria with many restrictions related to the past medical history, biologic use prior to enrollment, and laboratory tests have led to further reduction in the number of eligible patients in clinical trials.^[18]^ Inclusion criteria usually specifies a disease activity in the moderate to severe range; however, exclusion criteria restrict the range of disease activity by limiting use of steroids or other immunosuppressive treatments.^[19,20]^ More medications have been approved for SLE and the standard of care treatment is evolving but inclusion/exclusion mostly remains the same, and as such more patients are excluded from studies.

Despite recent advances in phase 2 clinical trial design including adaptive designs and withdrawal of standard of care therapies, SLE clinical trial design has remained vastly unchanged.^[20]^ While phase 2/dose finding study design randomizes patients 1:1:1:1 (drug:drug:drug:placebo), phase 3 studies have most often randomized patient 1: 1 drug to placebo. Possibly, investigators and patients with higher disease activity are more comfortable enrolling in phase 2 trials given the higher chance of receiving study drug, as such phase 3 trials may discourage enrollment of sicker patients. Furthermore, non-inferiority or superiority studies with newly approved medications as the comparator may be needed to encourage enrollment in clinical trials.

## Investigator/Sponsor Challenges

At an investigator and site level, there are an insufficient number of investigators with experience in SLE management and clinical trials; as disease activity measures are complex and require expertise in SLE.^[20]^ Rheumatology training programs for clinicians focus on clinical training, and there are no formal programs for training SLE investigators. Additionally, site staff problems such as high turnover in coordinators and clinical research managers may further hinder clinical trial operations.^[21]^

Furthermore, the smaller size of phase 2 trials allows for easier monitoring at all levels. The global phase 3 trials extend to areas of the world with less resources, responsibilities are delegated to sites, investigators, and monitors with limited experience. Continued involvement of the sponsors in the operations and monitoring of clinical trials is essential to success.

## Phase 3 SLE Clinical Trials

### Ustekinumab

The Ustekinumab SLE trial was stopped early for futility, these data offer the possibility that outcome evaluations were plagued by errors as the placebo arm of the trial had a 56% response on the SLE Responder Index (SRI)-4 compared to 44% response in the treatment arm. Interestingly, when evaluating reduction in steroid dose 30% of the patients in the treatment arm were able to taper their steroid dose as opposed to only 24% of the patients in the placebo arm. The key secondary outcome measures were the same between treatment and placebo, swollen and tender joint counts, improved in 64% of the patients in the treatment arm and 66% in placebo. Even the Cutaneous Lupus Erythematosus (LE) Activity and Severity Index (CLASI) that performed well in the anifrolumab studies did not work, 40% of the treated patients *vs*. 56% of the patients in the placebo arm responded.^[7]^

### Baricitinib

The next trial (s) reviewed are the pair of disappointing phase 3 clinical trials for baricitinib in lupus. These the two pivotal phase 3 trials of baricitinib had similar study design, each included over 700 patients. Patients were randomized to receive either baricitinib 2 mg, 4 mg, or placebo. The Baricitinib for systemic lupus erythematosus: a double-blind, randomised, placebo-controlled, phase 3 trial (SLE-BRAVE) I study achieved a statistically significant difference in SRI-4 response between placebo and the 4 mg/day arm (placebo response 45.9%, baricitinib 56.7%). However, in the BRAVE II study, there were no differences in the treatment or placebo response rates (45.6% of patients in the placebo arm, 46.3% of the patients in the 2 mg arm, and 47.4% of the patients in a 4 mg arm achieved the SRI). When evaluating the differences between the BRAVE I and BRAVE II patient characteristics no trends were noted. The secondary end points were also not met in either of these studies; glucocorticoid reduction, achievement of low disease activity, changes in pain or fatigue were the same in the treatment and placebo arms. While these phase III data are being evaluated to better understand drivers of response, at a molecular level, the development of baricitinib for the treatment of lupus is no longer pursued.^[8,9]^

## Phase 2 SLE Clinical Trials

### Iberdomide

Iberdomide is a cereblon modulator promoting degradation of the transcription factors Ikaros and Aiolos, which affect leukocyte development and autoimmunity. In this study SLE patients were randomly assigned to receive oral iberdomide (at a dose of 0.45, 0.30, or 0.15 mg) or placebo. The primary end point at week 24 was SRI-4 response; the percentages of patients with an SRI-4 response were 54% in the iberdomide 0.45-mg group, 40% in the iberdomide 0.30-mg group, 48% in the iberdomide 0.15-mg group, and 35% in the placebo group. The primary endpoint was met, with a statistically significant SRI-4 response at week 24 for the high-dose group. Effects were greater in 2 biomarker-defined populations (Aiolos-High, Type 1 Interferon [IFN]-High), providing an opportunity for precision medicine. Supportive efficacy was observed across multiple secondary endpoints and Iberdomide was well tolerated, with an acceptable safety profile.^[22]^

### Deucravacitinib

This study assessed deucravacitinib, an oral, selective, allosteric inhibitor of Tyrosine Kinase (TYK) 2, in patients with active SLE; 362 patients were randomized to deucravacitinib 3 mg twice daily, 6 mg twice daily, 12 mg once daily, or placebo. At week 32, 34% of the patients treated with placebo compared to 58% with deucravacitinib 3 mg twice daily, 50% in the 6 mg twice daily, and 45% with 12 mg once daily (Odd Ratio 1.6 [95% CI: 0.8, 2.9]) achieved the primary endpoint SRI-4 response. Response rates were higher in deucravacitinib treated patients for British Isles Lupus Assessment Group-based Composite Lupus Assessment (BICLA), Cutaneous LE Disease Area and Severity Index (CLASI)-50, Lupus Low Disease Activity State (LLDAS), and joint counts. No safety concerns were noted.^[23]^

### Litifilimab

Litifilimab is an anti-BDCA2 antibody in development for the treatment of SLE. Plasmacytoid dendritic cells (pDCs) have been identified as the main IFN-producing cells in cutaneous lupus and SLE. Blood dendritic cell antigen 2 (BDCA2) is a type II, Ctype lectin receptor expressed on the cell surface of pDCs. Litifilimab is a humanized IgG 1 monoclonal antibody that recognizes BDCA2 that disables pDCs and inhibits production of IFN and other inflammatory mediators. The studies reviewed here represent an innovative trial design that evaluated the efficacy of the new treatment in patients with either cutaneous disease, or arthritis, and included a mandated steroid taper. The number of active joints was the primary efficacy end point for the first study, on average treatment decreased the number of active joints by 15 as opposed to placebo where the difference was 11.6 joints. The CLASI provided the endpoint in the other trial that involved only participants with cutaneous lupus; treatment with litifilimab was superior to placebo at 16 weeks in decreasing cutaneous activity. Larger and longer trials are needed to determine the safety and efficacy of litifilimab in the treatment of lupus.^[24,25]^

### Obexelimab

Obexelimab is an investigational, bi-specific, noncytolytic monoclonal antibody that binds CD19 and FcyRIIb to inhibit B cells, plasmablasts, and plasma cells. The trial featured and innovative trial design, patients with active, non-organ-threatening SLE received corticosteroid to improve symptoms, and as would be the norm in practice the ineffective immunosuppressants were withdrawn (≤10 mg/day prednisone equivalent and ≤400 mg/day hydroxychloroquine were allowed). 104 patients with improved disease activity were randomized 1:1 to obexelimab 5 mg/kg intravenously or placebo once every 2 weeks until Week 32 or loss of improvement (LOI). The primary endpoint, proportion of patients reaching Week 32 without LOI, did not reach statistical significance: 21/50 (42.0%) obexelimab-treated *vs*. 12/42 (28.6%) placebo-treated patients (*P* = 0.183). Time to LOI was increased in obexelimabtreated *vs*. placebo-treated patients. Obexelimab was associated with gastrointestinal infusion reactions but was generally safe and well-tolerated. Although the primary endpoint was not reached, secondary analysis showed that time to LOI was significantly increased in obexelimab-treated patients, and analysis of patient subsets defined by gene expression patterns at baseline suggests a responding subpopulation.^[26]^

### Cenerimod

Sphingosine-1-phosphate (S1P) receptor modulators have been approved for the treatment of multiple sclerosis and inflammatory bowel disease. S1P is a signaling molecule that plays a critical role in atherosclerosis, inflammation, immunity, and cell proliferation, S1P receptors are part of the G protein-coupled receptor superfamily, cenerimod as a potent, selective S1P1 receptor modulator that reduces circulating T-and B-cells and tissue availability of T-cells in SLE mice.^[27, 28, 29]^ In a phase 2 double-blind placebo-controlled trial, cenerimod 4 mg resulted in a 4.04 lower modified Systemic Lupus Erythematosus Disease Activity Index 2000 (SLEDAI-2K) (to exclude white blood cells [WBC] as low lymphocytes are part of the mechanism of action of cenerimod) score compared to-2.85 for placebo. In exploratory/post-hoc analyses at Month 6, cenerimod 4 mg demonstrated a greater treatment effect size in patients with higher baseline disease activity and high IFN-1 gene expression signature. Over 12 months, treatment-emergent adverse events were similar across groups.^[30]^

### Dapirolizumab (DZP)

Interactions between CD40 ligand-L (CD40L, CD154; mainly expressed on activated T-cells and platelets) and the CD40 receptor (expressed on antigen-presenting cells and B-cells) play a key role in the adaptive immune response and drive the pathophysiology of SLE. Inhibiting the CD40L/CD40 interaction has been effective in murine SLE and CD40L has been an attractive therapeutic target in human SLE. In this phase 2 study, participants were randomized to receive DZP 6/24/45 mg/kg *vs.* Placebo IV, every 4 weeks for 24 weeks. The primary objective on this study was to identify a dose–response relationship, based on the primary efficacy variable of week 24 BICLA responder rates. While the primary endpoint was not met (none of the pre-specified dose–response relationship models fit the observed BICLA response rates at week 24), the published data from the phase 2 study propose efficacy across multiple clinical and immunological measures of disease activity after 24 weeks compared to placebo and substantiated a large phase 3 study. Incidences of serious treatment-emergent adverse events were similar in the DZP groups and placebo groups.^[31]^ Other CD40L products are in development for lupus and LN.

## LN Phase 2

### Anifrolumab Renal

The study assessed the efficacy and safety of the type I IFN receptor antibody, anifrolumab, in patients with active, biopsy-proven, Class III/IV lupus nephritis. This phase 2 double-blinded study randomized 147 patients (1:1:1) to receive monthly intravenous anifrolumab basic regimen (BR, 300 mg), intensified regimen (IR, 900 mg ×3 doses, 300 mg thereafter) or placebo, alongside standard therapy (oral glucocorticoids, mycophenolate mofetil). The study did not meet its primary endpoint, change in baseline 24-hour urine protein-creatinine ratio (UPCR) at week 52 for combined anifrolumab versus placebo groups. Numerically more patients treated with anifrolumab IR *vs.* placebo achieved complete renal response (CRR, 45.5% *vs.* 31.1%). Incidence of herpes zoster was higher with combined anifrolumab *vs.* placebo (16.7% *vs.* 8.2%). Incidence of serious adverse events was similar across groups.^[32]^

### Obinutuzumab Renal

B-cells can be selectively targeted via CD19, CD20, and CD22. Randomized trials of the anti-CD20 antibodies rituximab ^[33,34]^ and ocrelizumab ^[35]^ failed to show significant benefit in lupus nephritis, obinutuzumab, a humanized type II anti-CD20 monoclonal antibody that induces more potent B-cell depletion, has been investigated in a successful phase 2 trial for the treatment of lupus nephritis.^[36]^ Patients with LN receiving mycophenolate and corticosteroids were randomized to obinutuzumab 1000 mg or placebo on day 1 and weeks 2, 24 and 26, and followed through week 104. CRR was greater with obinutuzumab at week 52 (22 [35%] *vs.* 14 [23%]) with placebo; and at week 104 (26 [41%] *vs.* 14 [23%]). Improvements in other renal response measures, serologies, estimated glomerular filtration rate and proteinuria were also greater with obinutuzumab. Obinutuzumab was not associated with increases in serious adverse events, serious infections, or deaths.^[24]^ A phase 3 study for LN finished enrollment and the non-renal study is in full swing.

## Phase 1 SLE trials

### Chimeric antigen receptor (CAR) T cells

CAR T-cells are an engineered cell therapy products that combine B-cell antibody-based antigen recognition with T-cell cytotoxicity. Cytotoxic CD8^+^ CD19 CAR T-cells designed to deplete B-cells have produced promising results in several autoimmune disease indications, including SLE. Compared to monoclonal antibodies, CAR T-cells propose several advantages as they are long-lived cells that can multiply, traffic to the lymphoid tissues or target organs and develop into memory populations that can prevent the re-emergence of pathogenic lymphocytes. In SLE, some promising results were shown with CD19 targeted CAR T-cell therapy in severe refractory diseases. A small series of 5 patients, refractory to several immunosuppressive treatments, achieved SLE drug remission 3 months after the CD19 CAR T-cell administration without any toxicity signal.^[37]^ The five patients with SLE (four women and one man) had a median age of 22 years, median disease duration of four years and SLEDAI of 16. Autologous T-cells from patients with SLE were transduced with a lentiviral anti-CD19 CAR vector, expanded, and reinfused at a dose of 1 × 10^6^ CAR T-cells per kg body weight into the patients after lymphodepletion with fludarabine and cyclophosphamide. CAR T-cells expanded in vivo, led to deep depletion of B-cells, improvement of clinical symptoms and normalization of laboratory parameters including seroconversion of anti-double-stranded DNA antibodies. Definition of remission in SLE (DORIS) Remission was achieved in all five patients after 3 months and maintained during longer follow-up (median of 8 months after CAR T-cell administration) and even after the reappearance of B-cells. Reappearing B-cells were naïve and showed non-class-switched B-cell receptors. CAR T-cell treatment was well tolerated with only mild cytokine-release syndrome. These data suggest that CD19 CAR T-cell transfer is feasible, tolerable and highly effective in SLE. Several phase one studies are in progress to further define the role of CAR T therapy in SLE (Bristol Myers Squibb, Cabaletta, Kyverna).

## Conclusion

The management and prognosis of SLE has profoundly changed as new therapies became available.^[38]^ The recent European Alliance of Associations for Rheumatology (EULAR) guidelines for the treatment of SLE propose earlier use of biologics to decrease reliance on steroids and prevent damage. With a large pipeline of targeted treatments that showed efficacy in phase 2 studies, currently in ongoing phase 3 studies, we hope that the treatment armamentarium of SLE will soon include several of these new drugs. However, the need for new targeted treatments and new therapeutic strategies remain significant challenges in SLE therapy.^[39,40]^ The next task will be personalized treatment both to the clinical manifestations and pathophysiology of each patient.
